# Diabetic Retinopathy Is Strongly Predictive of Cardiovascular Autonomic Neuropathy in Type 2 Diabetes

**DOI:** 10.1155/2016/6090749

**Published:** 2016-02-03

**Authors:** Chih-Cheng Huang, Jong-Jer Lee, Tsu-Kung Lin, Nai-Wen Tsai, Chi-Ren Huang, Shu-Fang Chen, Cheng-Hsien Lu, Rue-Tsuan Liu

**Affiliations:** ^1^Department of Neurology, Kaohsiung Chang Gung Memorial Hospital, Chang Gung University College of Medicine, Kaohsiung 83301, Taiwan; ^2^Department of Ophthalmology, Kaohsiung Chang Gung Memorial Hospital, Chang Gung University College of Medicine, Kaohsiung 83301, Taiwan; ^3^Department of Biological Science, National Sun Yat-Sen University, Kaohsiung 80424, Taiwan; ^4^Department of Neurology, Xiamen Chang Gung Memorial Hospital, Xiamen, Fujian, China; ^5^Division of Metabolism, Department of Medicine, Kaohsiung Chang Gung Memorial Hospital, Chang Gung University College of Medicine, Kaohsiung 83301, Taiwan

## Abstract

A well-established, comprehensive, and simple test battery was used here to re-evaluate risk factors for cardiovascular autonomic neuropathy (CAN) in type 2 diabetes. One hundred and seventy-four patients with type 2 diabetes were evaluated through the methods of deep breathing and Valsalva maneuver for correlation with factors that might influence the presence and severity of CAN. The Composite Autonomic Scoring Scale (CASS) was used to grade the severity of autonomic impairment, and CAN was defined as a CASS score ≥2. Results showed that nephropathy, duration of diabetes, blood pressure, uric acid, and the presence of retinopathy and metabolic syndrome significantly correlated with the CASS score. Age may not be a risk factor for diabetic CAN. However, the effects of diabetes on CAN are more prominent in younger patients than in older ones. Diabetic retinopathy is the most significant risk factor predictive of the presence of CAN in patients with type 2 diabetes.

## 1. Introduction

Cardiovascular autonomic neuropathy (CAN) is one of the most clinically relevant complications of diabetes. The risk of developing CAN in diabetes depends on several factors, the most intuitive and most well-established of which is chronic hyperglycemia, including the duration and glucose level. Old age, nephropathy, and vascular risk factors such as hypertension and dyslipidemia have also been associated with increased severity of CAN [1–7]. Identifying the risk factors of CAN is essential in providing important clues to its etiologies and can help physicians determine treatment guidelines.

The prevalence of CAN among patients with diabetes varies widely in different reports, perhaps due to different patient groups (different ages and different durations of diabetes), different tests used, and different diagnostic criteria [5, 6]. Although there are no uniform criteria or staging for diagnosing CAN, advances in autonomic laboratory testing in the past decades, especially with the introduction of noninvasive beat-to-beat blood pressure (BP) recording by Finapres [8], have greatly improved the sensitivity and specificity of evaluations of cardiovascular autonomic function [9]. The American Academy of Neurology has published a position paper on autonomic function tests [10]. The autonomic tests used by previous studies have significant limitations. For example, most of them focused on cardiovagal function, whereas adrenergic function was either omitted or evaluated simply by BP changes with postural change or handgrip [1, 2, 7, 11, 12]. Such methods may have limited sensitivity and specificity according to evidence-based assessment [10]. Furthermore, in some studies, absolute cut-off values are used to define autonomic “abnormalities.” Thus, the confounding effects of age and sex are not eliminated.

The present study evaluated cardiovascular autonomic functions, including both cardiovagal and adrenergic functions, by using simple, time-saving, and well-established methods. Factors that might influence the presence and severity of CAN in patients with type 2 diabetes were also assessed. Lastly, the association between these risk factors and CAN was re-evaluated. The successful translation of these approaches to the clinics enables not only the prediction of outcome but also the assessment of the impact of factors on the therapeutic efficacy of patients with diabetes.

## 2. Patients and Methods

### 2.1. Inclusion and Exclusion Criteria

This cross-sectional study evaluated 174 patients with type 2 diabetes from the outpatient diabetes clinic at Kaohsiung Chang Gung Memorial Hospital between April 2011 and July 2011.

Patients were excluded if they had the following: (1) suffered from moderate-to-severe heart failure (NYHA class III and IV); (2) had any type of arrhythmia that prevented the analysis of heart rate variability, or pacemaker implantation due to any cause; (3) had neoplastic disorders; (4) had degenerative disorders known to affect the autonomic system, such as Parkinson's disease, diffuse Lewy-body disease, multiple system atrophy, and pure autonomic failure; or (5) had a history of major stroke (brain stem or large hemispherical lesions).

### 2.2. Study Protocol

The hospital's Institutional Review Committee on Human Research approved the study protocol, and all of the study subjects provided informed consent.

Each patient participated in a detailed interview regarding their personal disease and a physical examination that included measurements of height, weight, and waist circumference. All of the subjects then underwent an autonomic survey, including deep breathing and Valsalva maneuver (VM) tests, as described by Low [13].

### 2.3. Assessment of Cardiovascular Autonomic Function

Heart rate (HR) was derived from continuously recorded standard three-lead ECG (Ivy Biomedical, model 3000; Branford, CT). Arterial BP was continuously measured at the finger by using beat-to-beat photoplethysmographic recordings (Finometer Pro, Ohmeda; Englewood, OH). Parameters of HR response to deep breathing (HR_DB) and Valsalva ratio (VR) were obtained through tests computed by Testworks (WR Medical Electronics Company, Stillwater, MN). To quantify the degree of dysfunction, the measures of HR_DB and VR were transformed into normal deviates (NDs) by using the Neuropcentiles software (WR Medical Electronics Company) [14] and denoted by *Z*_HR_DB_ and *Z*_VR_, respectively.

The severity of CAN was assessed by using the cardiovagal and adrenergic subscores of the Composite Autonomic Scoring Scale (CASS) [15]. However, the scale was modified for the adrenergic subscore because the 5-minute head-up tilt test was not performed in the current study. Thus, the CASS version used here allotted 3 points instead of 4 for the adrenergic domain (Table 1).

### 2.4. Assessment of Risk Factors

The parameters evaluated were age, duration of diabetes, microvascular complications of diabetes (retinopathy and nephropathy), diabetic control (glycohemoglobin, HbA1c), associated medication (i.e., insulin, diuretics, beta-blockers, angiotensin-converting enzyme inhibitors/angiotensin II receptor blockers [ACEI/ARB], and calcium channel blockers [CCB]), inflammatory condition (hsCRP), body mass index (BMI), waist circumference, and biochemical data, including total cholesterol, triglycerides, high-density lipoprotein cholesterol (HDL-C), low-density lipoprotein cholesterol (LDL-C), uric acid, serum creatinine, and estimated glomerular filtration rate (GFR), which was calculated by using the modified Diet and Renal Disease equation. Albuminuria was determined by measuring the urinary albumin-to-creatinine ratio (UACR) in a spot urine test.

Retinopathy was determined through fundus photography by an experienced ophthalmologist (J.-J. Lee) who was blinded to the autonomic test results. Diabetic retinopathy (DR) was classified as one of the following three stages: stage 0: no apparent retinopathy (equivalent to the scale of Early Treatment of Diabetic Retinopathy Study [ETDRS] level 10); stage 1: nonproliferative diabetic retinopathy (NPDR; ETDRS level 20–55); and stage 2: proliferative diabetic retinopathy (PDR, ETDRS level >61) [16].

Moreover, hypertension was defined as a systolic BP > 140 mmHg and/or a diastolic BP > 90 mmHg, or being under antihypertensive treatment. Metabolic syndrome was defined as meeting at least two of the following criteria: (1) waist circumference >90 cm for men and >80 cm for women, (2) serum triglyceride level ≥150 mg/dL or being under drug treatment for elevated triglycerides, (3) serum HDL-C level <40 mg/dL in men and <50 mg/dL in women, or being under drug treatment for low HDL-C, and (4) elevated BP (systolic BP ≥ 130 mmHg and/or diastolic BP ≥ 85 mmHg, or a previous diagnosis of hypertension).

### 2.5. Statistical Analyses

Data are expressed as mean ± SD or median (interquartile range) for continuous variables and as median (minimum, maximum) for ordinal variables. Associations between measurements were evaluated with Pearson correlation tests for normally distributed continuous data or with the Spearman nonparametric test for continuous data with skewness or for ordinal data. The chi-square test was used for analyses of dichotomous variables. Logistic regression analysis with the forward conditional method was used to identify the odds ratio (OR) of risk factor. Statistical significance was set at *p* < 0.05. All statistical analyses were conducted by using the IBM SPSS software package, version 17 (IBM, Inc., Armonk, NY).

## 3. Results

### 3.1. General Characteristics and Autonomic Function of Patients with Diabetes

**Figure 1:**
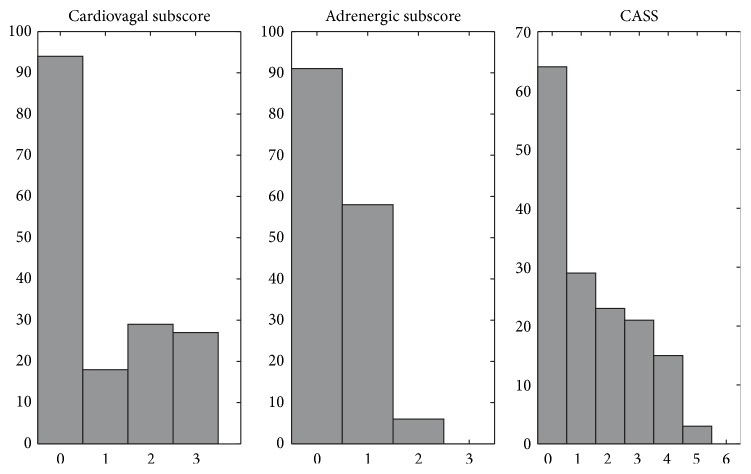
Histograms of cardiovascular autonomic scores, including cardiovagal and adrenergic subscores, and cardiovascular CASS score of the study patients. CASS: Composite Autonomic Scoring Scale.

Of the 174 (117 men, 57 women) patients diagnosed with type 2 diabetes, 56 were administered insulin therapy. Their demographic characteristics and biochemical and autonomic parameters are listed in Table 2. Most of them had hypertension (153/174) and metabolic syndrome (136/174). The histograms of cardiovagal and adrenergic subscores and CASS are shown in Figure 1. On the histogram, the total valid number of adrenergic subscores and CASS is <174 because subjects who had suboptimal Valsalva effort (expiratory pressure <30 mmHg or duration <10 s) and undetermined scores were not included in the analysis.

Of the 159 patients with a valid CASS score, 41.5% (66/159) had CAN, which was defined as a minimum score of 1 in both the cardiovagal and adrenergic domains or a minimum score of 2 in one domain [5, 17]. In other words, patients with a CASS score ≥2 were defined as having CAN.

### 3.2. Risk Factors Associated with CAN

The CAN group was younger (60.7 ± 9.4 versus 64.8 ± 8.3 years, *p* = 0.005) and had higher UACR levels (0.14 versus 0.05, *p* = 0.001), compared with the non-CAN group (Table 3). The CAN group had a significantly higher stage of DR than did the non-CAN group (*p* < 0.001). The proportion of patients using insulin and diuretics were borderline higher in the CAN group (*p* = 0.050 and 0.044, respectively). There was no difference between the two groups with respect to sex, BMI, waist circumference, diabetic profile (including duration of diabetes and HbA1c level), lipid profile, GFR, uric acid, or hsCRP level. Although the CAN group had a higher prevalence of metabolic syndrome and higher BP, the differences were not statistically significant.

Statistical analysis of the differences between clinical manifestations and laboratory data between the two patient groups revealed significant findings for the following parameters: age (*p* = 0.005), UACR (*p* = 0.001), insulin usage (*p* = 0.05), and stage of retinopathy (*p* < 0.001). The significant univariate factors and possible confounding factors used in stepwise logistic regression included age, UACR, insulin usage, and retinopathy stage. After analysis of all the above-mentioned variables, only age and retinopathy stage were independently associated with the presence of CAN. Each reduction of a year of age increased the rate of CAN by 5% (*p* = 0.012, adjusted OR = 0.95, 95% CI = 0.90–0.99). The patients with stage 1 and stage 2 DR had a higher risk of CAN than did those without CAN (stage 0) by adjusted ORs of 2.73 and 11.19, respectively (Table 4).

### 3.3. Associations between Risk Factors and Autonomic Parameters

An analysis of the association between risk factors and individual autonomic parameters and scores (Table 5) revealed that the correlations of age with HR_DB, cardiovagal subscore, and CASS were significant. The duration of diabetes and systolic BP both significantly correlated with *Z*_VR_. As for the indicators of nephropathy, UACR significantly correlated with all autonomic parameters and scores, whereas GFR correlated with only some of them. There were significant correlations between UA and adrenergic subscores and between UA and CASS. The presence of DR and metabolic syndrome was significantly associated with lower *Z*_HR_DB_ and *Z*_VR_.

## 4. Discussion

Although cardiovascular autonomic reflex tests [10] have been accepted as the gold standard for the evaluation of autonomic function, no unanimous criteria for diagnosis of CAN have been adopted to date [5, 6]. This study intended to introduce a simple and time-saving test battery that can be applied to clinical practice, rather than just study purposes. These test procedures can be performed within 10–15 min, and both cardiovagal and adrenergic functions can be evaluated. The deep breathing test was previously recommended as an optimal test for cardiovagal function [10]. VM tests can detect adrenergic failure with greater sensitivity than can orthostatic BP recordings [9]. Furthermore, these methods have well developed age normative data and a corresponding scaling score for the degree of severity.

The prevalence of CAN in the study patients was 41.5% (66/159). Among them, 44 (66.7%) had a CASS score of 2 or 3. The prevalence of CAN in previous reports varied widely from 2.5 to 50% [6]. Factors that influence the prevalence of CAN include the diagnostic criteria used, patient age, and the duration of diabetes. However, our results are consistent with findings in most previous reports in the aspect that autonomic neuropathy is common in diabetes, although it tends to be of a mild severity [3, 17].

Surprisingly, the CAN group was younger than the non-CAN group. There were strongly significant correlations between age and *Z*_HR_DB_ and between age and the cardiovagal subscore. However, the correlation coefficient for the correlation between age and *Z*_HR_DB_ was positive, whereas that for the correlation between age and the cardiovagal subscore was negative, indicating that older age is associated with larger ND (and less abnormality). In addition, in the multivariate analysis, the OR for CAN was 0.94 for age, suggesting that CAN is less likely to occur in older age. These findings seem to contradict those in several previous reports showing that age is a risk factor for CAN [1, 18]. Those studies used absolute cut-off values as the definition of “abnormal” autonomic function and thus the confounding effects of age were not eliminated. However, according to the report of expert panels on consensus of diabetic neuropathy in Toronto, age normative values should be used in testing cardiovascular autonomic function since age is the most important cofounding factor [5, 6]. In fact, in our data, if the original measures were used instead of the *Z*-scores for association analyses, the correlation coefficient would have been negative. The method of using percentiles or NDs (*Z*-scores) to express the degree of test abnormalities was introduced by Dyck et al. [14]. This statistical method gives useful information about dysfunction or disease even when results fall within the range of normal values. In addition, the age (and gender) effects can be eliminated by using the transformed *Z*-scores. Because the current study focused on CAN in diabetes, the effects of normal aging on the autonomic system had to be excluded. Thus, using *Z*_HR_DB_ and *Z*_VR_ was more appropriate than using HR_DB and VR in the analyses. The results here are not unique since the findings by O'Brien et al. are similar [4]. Using age-adjusted normal ranges rather than absolute cut-off values in this study, abnormal autonomic scores correlated significantly with the duration of diabetes but not with age. In addition, the frequency of abnormal autonomic scores was greatest in the group aged 40–49 years rather than in the oldest group. The Toronto consensus panel in 2009 did not include old age as a risk factor of CAN [6]. Furthermore, two previous studies found that a younger age of onset is a risk factor for diabetic retinopathy [19] and nephropathy [20]. Considering that diabetic retinopathy, nephropathy, and neuropathy share a common mechanism, that is, microvasculopathy, our results may not be unexpected. We suggest that the duration of undiscovered type 2 diabetes, which may be longer in younger patients, may contribute, at least partially, to the phenomenon. Overall, the current results suggest that the effects of diabetes on cardiovascular autonomic function are more obvious in younger patients than in older ones; however, it is impossible to demonstrate in detail the influence of age on diabetic CAN through only a cross-sectional study. Elucidating the real effects of age on diabetic CAN warrants further longitudinal cohort studies.

According to our data, DR is a strong predictor for CAN. The importance of such a finding has not been sufficiently stressed although the correlation between CAN and retinopathy has been mentioned in some reports [2, 21, 22]. Schmid et al. found that proliferative DR was related to CAN in type 2 diabetes [22]. However, their case number was limited (*n* = 17 and 18 for non-CAN and CAN groups, respectively) and thus the results may be less compelling. The results here corroborate such findings and suggest that fundus photography may be an alternative to autonomic function testing in hospitals where facilities for the latter test are unavailable, because of the robust ORs (2.73 and 11.19 for stage 1 and stage 2, respectively).

The correlation between nephropathy and CAN has been reported in several studies [23–25]. The results are consistent with those of previous findings. Although in multivariate logistic analysis, UACR is not a significant predictor for CAN, there are strong correlations between UACR and each autonomic parameter in bivariate analyses. Albuminuria is often considered as a manifestation of microvasculopathy. The strong correlation between UACR and autonomic function again supports the notion that microvasculopathy plays an important role in CAN. Although albuminuria and decreased GFR are both considered indicators of nephropathy, the results here show that the correlation between CAN and UACR may be stronger than that between CAN and GFR. A previous study by Sterner et al. showed that a significant correlation between albuminuria and low GFR only exists in patients with type 1 diabetes but not in those with type 2 [26]. The more complicated and heterogeneous pathophysiology of reduced GFR in type 2 diabetes compared to that in type 1 diabetes may explain such findings.

Vascular risk factors such as hypertension and dyslipidemia have been associated with CAN. The data here shows that systolic BP significantly correlates with *Z*_VR_; however, the interaction is likely to be reciprocal rather than unidirectional. Although hypertension may contribute to the existence of CAN, causing the decreased *Z*_VR_ that usually suggests blunted baroreflex sensitivity, patient with blunted baroreflex sensitivity tend to be hypertensive [27]. There was no significant correlation between lipid profile and cardiovascular autonomic function in this study.

The effects of UA on autonomic function or cardiovascular function remain controversial [28, 29], although there were significantly positive correlations between UA level and adrenergic subscore in this study. There was a borderline difference in insulin use between the CAN and non-CAN groups, which may be explained by confounding factors. The patient group with insulin treatment tended to have longer DM duration, higher HbA1c, and poorer renal function.

This study has some limitations. First, the prevalence of CAN in such patients cannot reflect the real conditions of general patients with diabetes. Patients with better compliance tend to be recruited in studies; hence, these patients have relatively good serum glucose control. The HbA1c value of the patients in this study was 7.2 ± 0.9. This narrow HbA1c spectrum may explain why statistical analyses fail to show any significant correlation between HbA1c and autonomic parameters. Furthermore, the medication effects on autonomic function tests were not eliminated in this study. Beta-blockers, CCBs, and diuretics are likely to influence the autonomic test results. Due to ethical considerations, these drugs were not stopped before the tests. Fortunately, the effects did not seem to be obvious and there was only a borderline significant difference in diuretic use between the CAN and non-CAN groups.

In conclusion, retinopathy is the most significant risk factor in predicting the presence of CAN in patients with type 2 diabetes. Old age may not be a risk factor for diabetic CAN. On the contrary, the effects of diabetes on CAN are more prominent in younger patients than in older ones.

## Figures and Tables

**Table 1:** Modified Composite Autonomic Scoring Scale (subscores in cardiovagal and adrenergic domains).

Cardiovagal	
0	Normal
1	HR_DB mildly reduced but >50% of minimum
2	HR_DB reduced to <50% of minimum or HR_DB + VR reduced
3	Both HR_DB and VR reduced to <50% of minimum

Adrenergic	

0	Normal
1	Early phase II reduction >20 but <40 mmHg MBP (30–40 if >50 years)
	Late phase II does not return to baseline
	Pulse pressure reduction to ≤50% of baseline
2	Early phase II reduction >40 mmHg MBP
3	Early phase II reduction >40 mmHg + absent late phase II and phase IV

HR_DB: heart rate response to deep breathing; VR: Valsalva ratio; MBP: mean blood pressure.

**Table 2:** Characteristics and biochemical data of patients with type 2 diabetes.

Characteristics	Mean ± SD [median (IQR)]
Age (year)	63.8 ± 9.2
Male/female	117/57
Body weight (kg)	69.8 ± 12.2
Body height (cm)	162.4 ± 8.0
Body mass index (kg/m^2^)	26.2 ± 3.7
Waist circumstance (cm)	93.2 ± 10.6
Duration of diabetes (year)	11.9 ± 7.0

HbA1c (mmol/mol) (NGSP, %)	55 ± 10 (7.2 ± 0.9)
GFR (mL/min/1.73 m^2^)	60.4 ± 29.1
UACR (mg/mg)	0.10 [0.02, 0.38]
hsCRP (mg/L)	1.00 [0.44, 2.3]
UA (mg/dL)	7.3 ± 2.0
Cholesterol (mg/dL)	153.2 ± 29.5
LDL-C (mg/dL)	74.1 ± 26.3
HDL-C (mg/dL)	52.3 ± 13.6
Triglycerides (mg/dL)	114.0 [80.8, 168.0]

SBP (mmHg)	138.8 ± 19.1
DBP (mmHg)	74.2 ± 10.5

HR_DB (beats/min)	7.2 ± 4.6
VR	1.29 ± 0.18
*Z*_HR_DB_	−1.04 ± 1.06
*Z*_VR_	−1.83 ± 0.78
Cardiovagal subscore	0 [0, 3] [min, max]
Adrenergic subscore	0 [0, 2] [min, max]
CASS	0 [0, 5] [min, max]

*n*: valid case number; SD: standard deviation; IQR: interquartile range; HbA1c: glycohemoglobin; GFR: glomerular filtration rate; UACR: urinary albumin-to-creatinine ratio; UA: uric acid; hsCRP: high-sensitive C-reactive protein; LDL-C: low-density lipoprotein cholesterol; HDL-C: high-density lipoprotein cholesterol; SBP: systolic blood pressure; DBP: diastolic blood pressure; HR_DB: heart rate response to deep breathing; VR: Valsalva ratio; CASS: Composite Autonomic Scoring Scale.

**Table 3:** Demographic data between groups of CAN and non-CAN.

	Non-CAN (*n* = 93)	CAN (*n* = 66)	*p* value
Age (year)	64.8 ± 8.3	60.7 ± 9.4	0.005^*∗∗*^
Body mass index (kg/m^2^)	26.0 ± 3.6	26.3 ± 3.8	0.524
Waist circumstance (cm)	92.9 ± 10.6	92.9 ± 10.8	0.815
Duration of diabetes (year)	11.5 ± 6.7	12.8 ± 7.5	0.331
SBP (mmHg)	137.1 ± 20.1	141.0 ± 18.7	0.205
DBP (mmHg)	74.4 ± 11.3	74.5 ± 10.0	0.921
HbA1c (mmol/mol) (NGSP, %)	54 ± 9 (7.1 ± 0.8)	56 ± 12 (7.3 ± 1.1)	0.175
GFR (mL/min/1.73 m^2^)	63.1 ± 26.8	58.9 ± 31.9	0.120
UACR (mg/mg)	0.05 [0.01, 0.24]	0.14 [0.05, 0.57]	0.001^*∗∗*^
hsCRP (mg/L)	0.95 [0.44, 2.02]	1.05 [0.49, 2.30]	0.442
UA (mg/dL)	7.1 ± 1.8	7.6 ± 2.3	0.126
Cholesterol (mg/dL)	151.3 ± 32.8	155.7 ± 25.0	0.115
LDL-C (mg/dL)	72.7 ± 28.9	76.3 ± 23.8	0.112
HDL-C (mg/dL)	51.8 ± 12.6	54.7 ± 14.8	0.222
Triglycerides (mg/dL)	135.0 ± 85.0	123.5 ± 70.0	0.600

Sex (F/M)	26/67	24/42	0.263
Insulin	24/93	27/66	0.050^*∗*^
ARB/ACEI	72/93	54/66	0.678
Beta-blocker	24/93	27/66	0.055
CCB	34/93	29/66	0.410
Diuretics	46/93	44/66	0.044^*∗*^
Metabolic syndrome	66/93	55/66	0.059
Retinopathy^†^			**<**0.001^*∗∗*^

HbA1c: glycohemoglobin; GFR: glomerular filtration rate; UACR: urinary albumin-to-creatinine ratio; hs-CRP: high-sensitive C-reactive protein; UA: uric acid; LDL-C: low-density lipoprotein cholesterol; HDL-C: high-density lipoprotein cholesterol; ARB: angiotensin receptor blocker; ACEI: angiotensin-converting-enzyme inhibitor; CCB: calcium-channel blocker.

^*∗*^ *p* < 0.05; ^*∗∗*^ *p* < 0.01.

^†^Retinopathy was categorized into stages 0, 1, and 2.

**Table 4:** Logistic regression analyses of risk factors for cardiovascular autonomic neuropathy.

	Adjusted OR of CAN (95% CI)	*p* value
Age	0.95 (0.90–0.99)	0.012^*∗*^
UACR	1.24 (0.66–2.33)	0.504
Retinopathy		
Stage 1	2.73 (1.14–6.54)	0.024^*∗*^
Stage 2	11.19 (4.15–30.16)	**<**0.001^*∗*^

OR: odds ratio; CAN: cardiovascular autonomic neuropathy; CI: confidence interval; UACR: urinary albumin-to-creatinine ratio.

^*∗*^ *p* < 0.05.

**Table 5:** Univariate correlation analysis between individual risk factors and autonomic parameters/scores.

	*Z*_HR_DB_	*Z*_VR_	Cardiovagal subscore	Adrenergic subscore	CASS
Age	0.390^*∗∗*^	0.004	−0.371^*∗∗*^	0.158	−0.176^*∗*^
Body mass index	−0.107	−0.079	0.096	0.003	0.085
Waist circumstance	−0.071	−0.049	0.056	−0.077	0.029
Duration of diabetes	−0.017	−0.200^*∗*^	0.023	0.154	0.077
HbA1c	−0.089	−0.077	0.145	−0.022	0.069
SBP	−0.020	−0.205^*∗*^	0.051	0.148	0.081
DBP	0.007	−0.113	−0.039	0.013	−0.050
GFR	0.086	0.209^*∗*^	−0.048	−0.287^*∗∗*^	−0.153
UACR	−0.282^*∗∗*^	−0.287^*∗∗*^	0.289^*∗∗*^	0.225^*∗∗*^	0.326^*∗∗*^
hsCRP	−0.088	−0.153	0.093	0.106	0.102
UA	−0.090	−0.139	0.111	0.248^*∗∗*^	0.187^*∗*^
Cholesterol	−0.117	−0.078	0.122	0.028	0.073
LDL	−0.109	−0.063	0.111	0.045	0.092
HDL	−0.028	0.025	0.035	0.022	0.022
Triglycerides	0.029	−0.146	0.011	0.042	0.024

Retinopathy^†^	−0.429^*∗∗*^	−0.346^*∗∗*^	0.435^*∗∗*^	0.248^*∗∗*^	0.429^*∗∗*^
Metabolic syndrome	−0.095	−0.200^*∗*^	0.164^*∗*^	0.172^*∗*^	0.203^*∗*^

HbA1c: glycohemoglobin; SBP: systolic blood pressure; DBP: diastolic blood pressure; GFR: glomerular filtration rate; UACR: urinary albumin-to-creatinine ratio; hs-CRP: high-sensitive C-reactive protein; UA: uric acid; LDL: low-density lipoprotein; HDL: high-density lipoprotein.

^*∗*^ *p* < 0.05; ^*∗∗*^ *p* < 0.01.

^†^Retinopathy is categorized into stages 0, 1, and 2.
